# Transcriptome Profiling of Transposon-Derived Long Non-coding RNAs Response to Hormone in Strawberry Fruit Development

**DOI:** 10.3389/fpls.2022.915569

**Published:** 2022-06-16

**Authors:** Xi Chen, Chengdong Wang, Bing He, Zifan Wan, Yukun Zhao, Fengqin Hu, Yuanda Lv

**Affiliations:** ^1^School of Agronomy and Horticulture, Jiangsu Vocational College of Agriculture and Forest, Zhenjiang, China; ^2^Engineering and Technical Center for Modern Horticulture, Jurong, China; ^3^Key Laboratory of Tobacco Biology and Processing, Ministry of Agriculture, Tobacco Research Institute of Chinese Academy of Agricultural Sciences, Qingdao, China; ^4^Excellence and Innovation Center, Jiangsu Academy of Agricultural Sciences, Nanjing, China

**Keywords:** long non-coding RNA, transposon, strawberry, fruit ripening, ABA

## Abstract

Strawberry is an economically grown horticulture crop required for fruit consumption. The ripening of its fruit is a complex biological process regulated by various hormones. Abscisic acid (ABA) is a critical phytohormone involved in fruit ripening. However, little is known about the long non-coding RNAs (LncRNAs), especially transposon-derived LncRNA (TE-lncRNA), response to hormones during fruit ripening in octoploid strawberry. In the study, the transcriptome data of developing strawberry fruits treated with ABA and its inhibitor Nordihydroguaiaretic acid (NGDA) were analyzed to identify responsive LncRNAs and coding genes. A total of 14,552 LncRNAs were identified, including 8,617 transposon-derived LncRNAs (TE-LncRNAs), 412 LncRNAs (282 TE-LncRNAs), and 382 ABA-sensitive LncRNAs (231 TE-LncRNAs). Additionally, a weighted co-expression network analysis constructed 27 modules containing coding RNAs and LncRNAs. Seven modules, including “MEdarkorange” and “MElightyellow” were significantly correlated with ABA/NDGA treatments, resulting in 247 hub genes, including 21 transcription factors and 22 LncRNAs (15 TE-LncRNAs). Gene ontology enrichment analysis further revealed that ABA/NDGA-responsive modules, including LncRNAs, were associated with various metabolic pathways involved in strawberry fruit development and ripening, including lipid metabolism, organic acid metabolism, and phenylpropanoid metabolism. The current study identifies many high-confidence LncRNAs in strawberry, with a percentage of them being ABA pathway-specific and 22 hub-responsive LncRNAs, providing new insight into strawberry or other Rosaceae crop fruit ripening.

## Introduction

Strawberry (*Fragaria* × *ananassa*) is an octoploid *Rosaceae* species that is a vital horticultural crop grown worldwide (Edger et al., 2019). It was first cultivated and developed in France, where it has remained popular for more than a century due to its juicy, sweaty texture and pleasant aroma (Darrow, 1966). Strawberry fruit is prevalent in almost all the food markets in every corner around the world, and it is also an essential feedstock for producing jelly jam, and biscuits. Although strawberries cannot cope under harsh growth conditions or many abiotic stress factors, greenhouse or artificial climate chambers provide a favorable environment for its harvesting (Khoshnevisan et al., 2013). Therefore, strawberry is a non-climacteric fruit (Given et al., 1988) that has been widely cultured in many countries, and in some areas, it has even become the most important crop for local farmers (Lieten, 2005; Antunes and Peres, 2013; Zhang et al., 2014). The fruit is the most valuable part of this economic crop, and as a result, the scientific research on strawberry fruit development is significantly meaningful and economically beneficial (Wang et al., 2017). Thus, it has emerged as a significant scientific study subject in strawberry breeding and botany research.

Angiosperm plants have conserved mechanisms for developing fruits and regulating fruit ripening (Forlani et al., 2019). To date, there are some key gene encoding proteins that regulate this progression that has been discovered to be conserved in structure and function across different plant species such as *NACs* (Mitsuda and Ohme-Takagi, 2008; Xu et al., 2021), *YABBY* (Dinneny et al., 2005), and *AGAMOUS* (Itkin et al., 2009) families, etc. Fruit development and ripening have been shown to be influenced by the phytohormones, auxin, gibberellic acid (GA), abscisic acid (ABA), jasmonic acid (JA), and cytokinin (CK), as well as brassinosteroids (BR) and ethylene (ET) (Chen et al., 2020). Auxin and cytokines were the primary regulators of cell division and tissue expansion in the carpel during the early stages of fruit development, respectively (Pattison and Catalá, 2012). Later on, ABA and ethylene were also found to play central regulatory roles in the process of climacteric-fruit ripening, with ABA playing a role in the process of non-climacteric-fruit ripening (Chen et al., 2020). Strawberry is a typical non-climacteric fruit. Through the study on the diploid strawberry (landwood, *F. vessa*), it was found that auxin and gibberellic acid could activate the expression of a gene (FveCYP707A4a) encoding cytochrome P450 monooxygenase, which is a key enzyme in the bio-degradation of ABA during the fruit development process (Liao et al., 2018). On the other hand, ABA has the potential to activate the *FveNCED* gene, which encodes the rate-limiting enzyme of ABA biosynthesis, and this activity would be enhanced during the ripening stage of the fruit.

Many studies have been conducted to determine how the expression of coding genes changes during the ripening of strawberry fruits, and the results have revealed the presence of many important coding genes. However, LncRNA, especially transposon-derived LncRNAs (TE-LncRNA), is still not fully understood or even illustrated. After the introduction of new generation sequencing technology, the high-through transcriptome analysis has facilitated the discovery of LncRNA greatly (Lv et al., 2019; Chen et al., 2022; Liang et al., 2022). Recently, LnRNAs were predicted and verified in the transcriptome of *F. vesca* fruit in altering stages (Kang and Liu, 2015). Later, functional LnRNAs that could regulate *F. vesca* (diploid strawberry) fruit ripening were identified (Tang et al., 2021). These results implied that the LnRNAs are involved in the ripening process, which remains largely unknown.

In the study, we systemically identified LncRNAs response to exogenous ABA and NDGA under the developing fruit of octoploid strawberry (*F. ananassa*). The responsive LncRNAs and differentially expressed genes were analyzed and then linked into modules using the weighted co-expression network method. According to the findings of this study, the LnRNA profile of the octoploid strawberry was determined, as well as the transcriptomic deviation induced by the exogenous hormone, both in coding RNAs and in LncRNAs.

## Materials and Methods

### Plant Materials and Growth Conditions

Octoploid strawberry cultivar ‘Toyonoka’ was cultivated in a greenhouse with photoperiod and temperatures of 14-h light, 25°C/10-h dark, 20°C as described by Li et al. (2019). Fruits of 2-week after anthesis were injected with 150 μl of exogenous ABA (1 μM) solution when the CK is of mock injection with distilled water. Fruits were collected 5 days after the injection and immediately deep-frozen with liquid nitrogen for further qRT-PCR validation. Three independent biological replicates for each condition were performed. The germplasm was acquired from the USDA-ARS National Plant Germplasm System (NPGS) with permission for scientific research. Detailed information on the variety could be found at npgsweb.ars-grin.gov (accession: PI616632).

### RNA-Seq Data Processing and Transcript Assembly

As mentioned in Li et al. (2019), RNA-seq files were obtained and uncompressed from the SRA database (Accession: PRJNA338879) (Li et al., 2019). Fastp v0.20.0 (Chen et al., 2018) was used to clean the raw data, which contained sequencing adapters, low-quality bases, and too short reads (50 bp). Cleaned data was then mapped to the *Fragaria* × *ananassa* Camarosa genome (v1.0.a2)^1^ using STAR v2.7.9a (Dobin and Gingeras, 2016) with two-pass mode. The aligned reads were then assembled with the known transcript annotation (v1.0.a2) (Liu et al., 2021) by reference annotation-based transcript (RABT) assembly algorithm and generated a combined GTF-formatted annotated file using StringTie v2.1.5 (Pertea et al., 2016). Finally, the expression abundance of transcripts was quantified as counts with the above-updated annotation using featureCounts (Liao et al., 2014) and then normalized as the fragments per kilobase of transcript per million fragments mapped (FPKM) value by a custom script.^2^ For downstream gene expression analysis, only transcripts with an FPKM > 1 in at least three samples were considered.

### Computational Prediction and Transposon Annotation of Long Non-coding RNAs

To identify potential LncRNAs, a strict computational strategy was performed as described by Lv et al. (2019). First, all transcript sequences were extracted by gffread v0.12.2 program (Geo and Mihaela, 2020). Secondly, we employed three tools, including CPC2 and PLncPRO, to predict the coding potential of every transcript. Swissport and Pfam protein databases were selected for PLncPRO program. Default parameters were then performed CPC2 and PLncPRO. Finally, non-coding transcripts larger than 200 bp, with an FPKM > 1 and joint from two tools were considered candidate LncRNAs for further analysis. Additionally, the EDTA program was used to annotate transposon elements (TE) in octoploid strawberries (Ou et al., 2019). Coding sequences of octoploid strawberry were employed to purge gene sequences in the TE library. To avoid overmasking, coordinates specified compared with known gene position were also whitelisted from TE annotation. RepeatModeler (Flynn et al., 2020) is used to salvage some TEs such as SINE and LINE types left by structure-based scans.

### Differential Gene Expression Analysis

With the combined transcript annotation, DESeq2 (Love et al., 2014) was used to perform pairwise comparisons between conditional samples to find differentially expressed genes (DEGs). The following criteria were used to determine whether transcripts, including coding and non-coding RNAs, were differentially expressed: (I) the log2 fold change had to be greater than 1 and (II) the adjusted *p*-value from DESeq2 analysis had to be less than 0.05.

### Co-expression Network Analysis

To link the coding and non-coding RNAs, the WGCNA tool was used to generate a weighted co-expression network using the above count matrix under ABA and NDGA conditions (Pei et al., 2017). The DESeq2 package standardized the raw count expression matrix (Love et al., 2014). Co-expression correlation between coding and non-coding RNAs was then calculated using Pearson correlation with *r*^2^ ≥ 0.85. The normalized expression data from coding and non-coding RNAs were extracted to construct an unsigned co-expression network using the WGCNA package with a soft threshold = 10 (Pei et al., 2017). Module assignment of coding and non-coding RNAs was identified using Topological Overlap Matrix (TOM). Besides, additionally, correlations between modules and treatments were evaluated, and transcripts from modules with significant correlations were retrieved and displayed using the Cytoscape software (Saito et al., 2012). Furthermore, hub genes were defined as those with a module-trait correlation value greater than 0.8 and membership within modules greater than 0.8.

### Gene Ontology Enrichment Analysis

To infer the potential biological function of LncRNAs, coding transcripts in modules related to different were then performed for Gene Ontology (GO) enrichment analysis using the agriGO v2.0 toolkit (Tian et al., 2017). Significantly, over-represented GO terms were detected *via* Fisher’s exact test, and multi-test adjustment was made using the Yekutieli (FDR under dependency) method with a cutoff of FDR < 0.05. GO enriched results were combined and visualized by clusterProfiler (Yu et al., 2012).

### RNA Extraction and qRT-PCR Analysis

Total RNA was extracted using the RNAprep Pure Plant Kit (Tiangen, China) and cDNA was synthesized using PrimeScript RT Master Mix (Takara, Japan) according to kit instructions. PCR reactions were performed on the LightCycler 96 Real-Time PCR System (Roche, United States) using the SYBR Premix DimerEraser kit (Takara, Japan). Each PCR reaction was repeated three times, and relative expression levels of transcripts were calculated using the comparative Ct (ΔΔCt) method. qRT-PCR primers were designed using Beacon Designer 8 software and can be found in Supplementary Table 9.

## Results

### Updated Genome Annotation Based on RNA-Seqs Under Treatments

**FIGURE 1:**
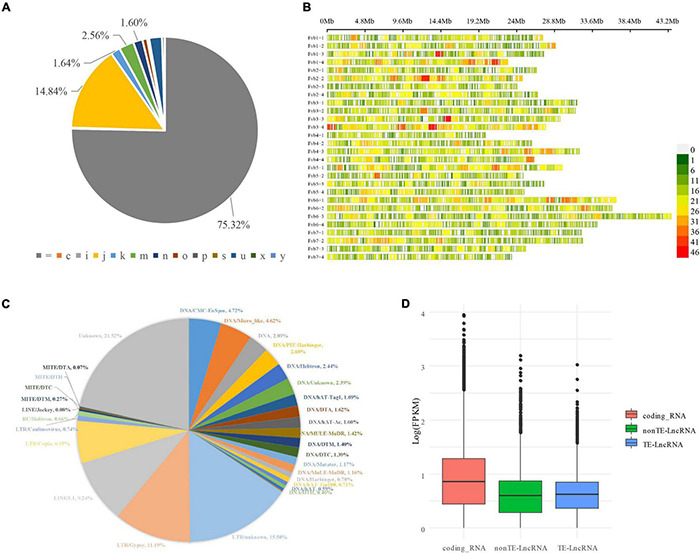
LncRNAs mining, identification, and expression level evaluation in strawberry developing fruit. **(A)** LncRNA distribution on the strawberry genome. The LncRNA distribution and density are demonstrated by the physical positions of chromosomes and coloration. The window wide is 1 M. **(B)** The result of StringTie analysis. The transcripts were categorified by StringTie software into different groups in different colors and marked with characters and symbols. For example, “ = ” complete as the reference transcript, exact match of intron cutoff; “i” fully contained within a reference intron; “u” intergenic or unknown. **(C)** Identifying transposon elements derived LncRNAs. **(D)** The Expression level evaluation on LncRNAs, TE-LncRNAs, and coding genes.

Fourteen RNAseq datasets from octoploid strawberry ripening fruit with treatments of phytohormone ABA and its inhibitor nordihydroguaiaretic acid (Li et al., 2019) were cleaned, mapped, and assembled with the RABT method. A total of 162,558 transcripts with 109,126 loci combined with known annotated transcripts were finally generated (Supplementary Table 1). By comparing with known annotation by gffcompare program (Geo and Mihaela, 2020), 3,595 novel transcripts were identified and located in the intergenic regions; 75.32% (122,445, “ = ”) transcripts were matched with the annotated genes perfectly; 14.84% (24,119, j) matching multiple exons with at least one junction connected; 1.60% (2,596, n) transcripts retained part of the intron; 0.16% (255) retained all the introns. There were also 2.21% (3,595, u) matching the intergenic region, and only 0.01% (24) transcripts were found to be located within the reference intron (Figure 1A and Supplementary Table 1).

### Identification and Characterization of Potential Long Non-coding RNAs

CPC2 and PLncPRO programs were used to identify potential LncRNAs from these candidate transcripts. Two state-of-the-art programs were engaged in proofreading the predicted LncRNAs. A total of 14,552 transcripts were identified as LncRNA (Supplementary Table 2). Only 1,029 LncRNA overlapped with known LncRNAs predicted by Edger et al. (2019). The distribution of the predicted LncRNA on octoploid strawberry chromosomes is shown in Figure 1B. Their FPKM value is highly varied in developing fruit. The transcripts with an FPKM value of more than 1 were deemed high-confidence stress-responsive transcripts. There hence were the transcriptions of 3,684 high-confidence expressed LncRNA detected in the study. Simultaneously, a total of 725 novel coding genes such as transposase-derived proteins FHY3/FAR1 (MSTRG.787, MSTRG.13323, MSTRG.39595, MSTRG.52515) were identified in developing fruit. By further annotating with transposon elements, we found 59.2% (8,617 of 14,552) LncRNAs were derived from transposon elements (TEs), and 2,074 with FPKM ≥ 1 were considered as high-confidence TE-LncRNAs (Supplementary Table 3). According to the TE family classification, the majority of TE-LncRNAs (530, 6.10%) were from the Copia family, followed by Gypsy (964, 11.1%) (Figure 1C). The details of TE annotation and TE-LncRNAs of the whole genome can be found in Supplementary Table 3. Besides, LncRNAs, especially TE-LncRNAs, showed a relatively lower expression level than coding genes, consistent with previous studies (Figure 1D; Lv et al., 2019).

### Abscisic Acid—and NDGA- Responsive Long Non-coding RNAs and Coding Genes

**FIGURE 2:**
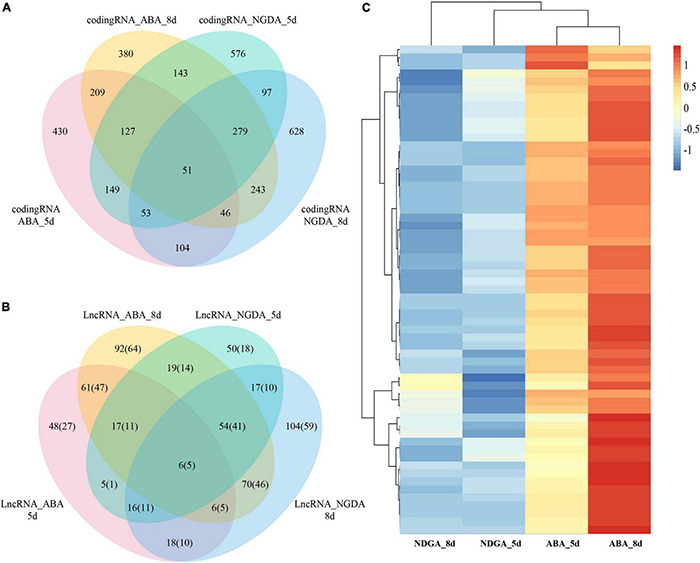
The Venn diagrams of 5- and 8-day ABA treatment and 5- and 8-day NGDA treatment. **(A)** The differentially expressed coding RNAs of ABA and NGDA treatment, and overlap between 5- and 8-day; **(B)** the differentially expressed LncRNAs of ABA and NGDA treatment, and overlap between 5- and 8-day; **(C)** the heatmap of the common ABA-responsive LncRNAs between ABA_5d and ABA_8d treatments.

According to the expression profile, differentially expressed genes were further analyzed by DESeq2. The results showed that ABA- and NDGA- treatment had significantly influenced the expression of LncRNAs and coding genes. In the ABA treatment, at 5- and 8- days after chemical injection, in comparison with control, there were 60/117 and 233/325 TE-LncRNA/LncRNAs, and 1,169 and 1,417 coding genes differentially expressed, respectively. Simultaneously, NDGA treatment at 5- and 8- days after injection, compared with control, there were 111/184 and 187/291 TE-LncRNA/LncRNAs, and 1,475 and 1,501 coding genes differentially expressed, respectively. As shown in VENN graphics (Figures 2A,B and Supplementary Table 4), for ABA, between 5- and 8-day, there were 90 LncRNAs (67 TE-LncRNAs) and 433 coding genes overlapped; between NDGA, 5- and 8-day, 93 LncRNAs (66 TE-LncRNAs) and 480 coding genes overlapped, respectively. Compared with NDGA treatment, 61 LncRNA (47 TE-LncRNAs) showed specific-responsive under ABA treatment across 5th and 8th day (Figure 2C and Supplementary Table 4).

### Co-expression Network of the Differentially Expressed Long Non-coding RNA

**FIGURE 3:**
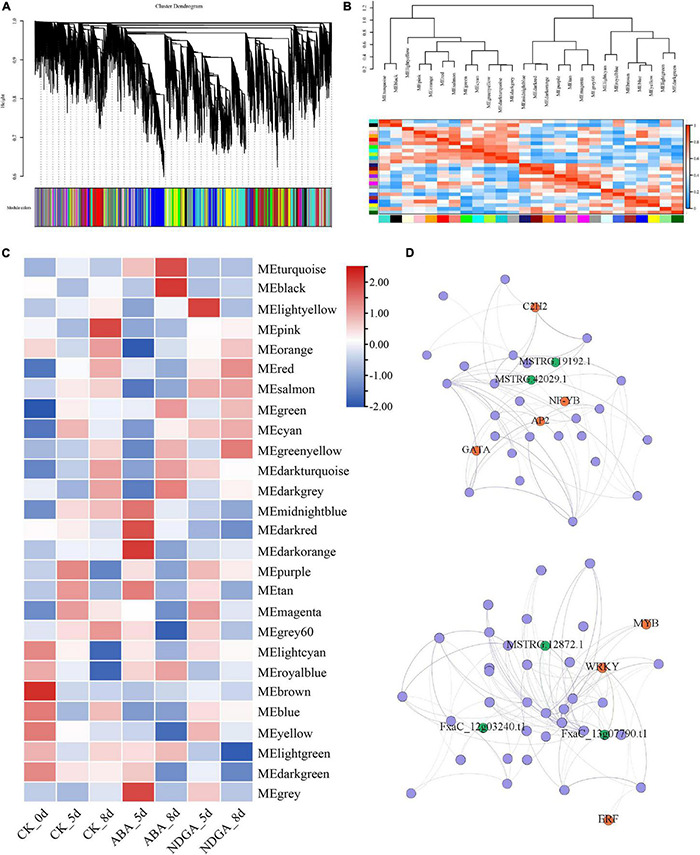
The co-expression network was constructed according to the differentially expressed LncRNAs and coding genes. **(A)** The clustering dendrogram with dissimilarity based on the topological overlap, together with assigned module colors; **(B)** the heatmap of correlation within co-expression modules; **(C)** the heatmap of correlation between co-expression modules and the treatments; **(D)** network illustration of co-expression module “MEdarkorange” and “MEblack.” The spots represent the node genes, which have high intramodular connectivities. Transcription factors are marked as orange, and the spots in blue are LncRNAs.

Predicting the biological role of LncRNAs in plant development and abiotic and biotic stresses remains a challenge. To investigate the potential functions of these ABA and NGDA treatment responsive LncRNAs, a well-developed computational algorithm WGCNA was employed to generate the weighted co-expression network based on the correlation between the expressing variation of LncRNAs and coding RNAs. The significantly correlated LncRNAs and coding genes were further constructed into the weighted co-expression network, from which a total of 27 modules were generated (Figure 3A and Supplementary Table 5). Furthermore, the correlation between the different modules, and the treatments, was also calculated. The correlation between the part of the modules is demonstrated by the heatmap and the dendrogram shown in Figure 3B. The heatmap in Figure 3C showed the correlation between a part of the modules with the treatments. In the figure, it was apparent that part of the modules positively correlated with the treatment, while the other part of them was negative.

In total, seven modules, including “MEdarkorange,” “MElightyellow,” “MEblack,” “MEturquoise,” “MEgrey60,” “MElightyellow,” and “MElightgreen” were significantly correlated with ABA/NDGA treatments, of which 247 hub genes including 21 transcription factors and 22 LncRNAs (15 TE-LncRNAs) were further identified (Figure 3C and Supplementary Tables 5, 6). For example, the modules “MEdarkorange” and “MElightyellow” showed the highest correlation with treatment ABA 5-day and NGDA 5-day, respectively (Figures 3C,D). The module “MEdarkorange” was constructed with 8 LncRNAs (4 TE-LncRNAs) and 54 coding genes (4 transcription factors). As shown in Figure 3D, the upper illustration demonstrated the regulation network of MEdarkorange. There were four TF coding genes (C2H2, NF-YB, AP2, and GATA) and LncRNAs (MSTRG19192.1 and MSTRG42029.1) playing a critical hub role in regulating the eigengenes. The lower one was the network of MElightyellow, which had the highest correlation with NGDA 5-day. There was 148 genes contained in this module, three TF coding genes (MYB, WRKY, and ERF), and five LncRNAs played an important hub role.

### Gene Ontology Enrichment

**FIGURE 4:**
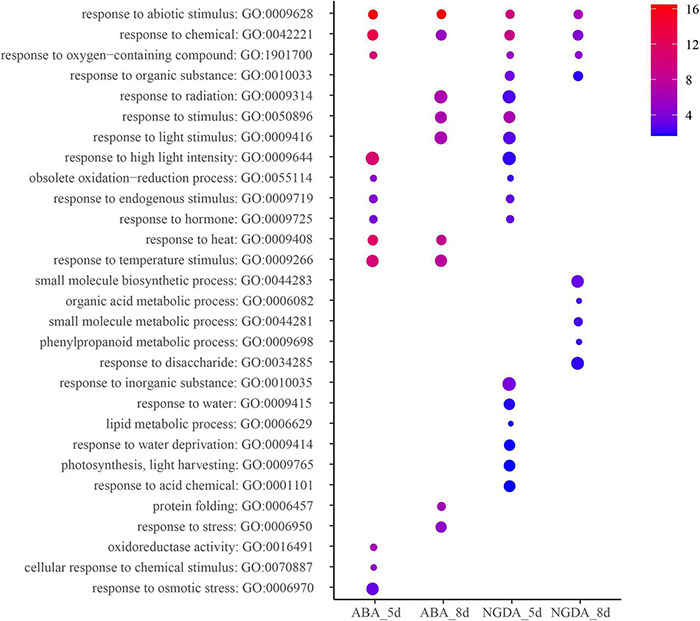
Gene ontology analysis of ABA/NDGA responsive modules. The heat map scale reflects the significant level of enrichment of GO terms. The red showed the most significant enrichment in statistics. Size of spot represents transcript numbers of terms.

The coding eigengenes in stress-responsive modules were analyzed by AgriGO V2 (Tian et al., 2017) to assign the enrichment. The coding eigengenes were selected from the module with the highest correlation with the treatment, and they were marked. The GO enrichment was demonstrated in Figure 4 and Supplementary Table 7. All of the treatments triggered the plant’s response to abiotic stimulus (GO:0009628) and response to chemicals (GO:0042221). The result also showed that there were several pathways relevant to fruit development enriched because of NGDA treatment for 8 days, such as small molecule biosynthetic process (GO: 0044283), organic acid metabolic process (GO: 0006082), small molecule metabolic process (GO:0044281), phenylpropanoid metabolic process (GO:0009698), and response to disaccharide (GO:0034285). The ABA and NGDA treatment could also influence the pathways concerned with light (GO:0009416, GO:0009644) and photosynthesis (GO:0009765). On the other hand, multiple pathways by which plant response to abiotic stress was significantly enriched, including response to heat (GO:0009408), response to water deprivation (GO:0009414), response to water (GO:0009415), response to osmotic stress (GO:0006970), etc.

### Evolutionary Conservation Analysis of Long Non-coding RNAs

The evolutionary conservation of identified octoploid strawberry LncRNAs was investigated among three related Rosaceae species: *Prunus salicina* (plum), *Malus domestica* (apple), and *Potentilla micrantha*. The homology analysis of 14,552 LncRNAs was carried out by Megablast with *E*-value ≤ 1e-10. As a result (Supplementary Table 8), 477(3.28%), 488(3.35%), and 3,760(25.8%) LncRNAs showed similarity with *P. salicina* (plum), *M. domestica* (apple), and *P. micrantha* genome. Besides, 268 LncRNAs were common among three species. These results suggested that the homology of LncRNAs was relatively consistent with phylogenetic distance and had lower conservation compared with coding RNAs.

### qRT-PCR Validation of the Long Non-coding RNA

**FIGURE 5:**
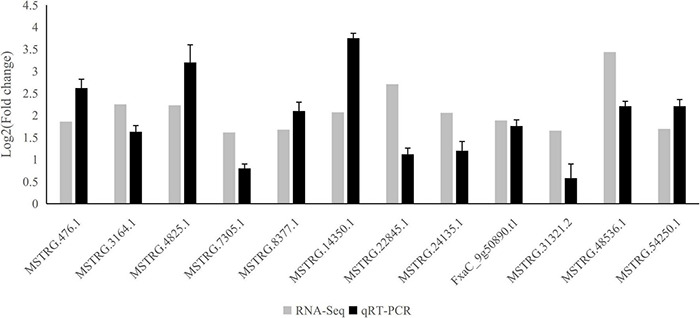
qRT-PCR validation of differentially expressed lncRNAs. The qRT-PCR histogram for each locus represents the mean ± standard error (SE) of three independent biological replicates, and the qRT-PCR was compared to fold-change data inferred from RNAseq data.

To validate the reliability of ABA-responsive LncRNAs, we performed an ABA-treatment experiment on strawberries (see section “Materials and Methods”), and their RNA was extracted. We then subjected the samples to quantitative real-time PCR (qRT-PCR) to compare expression changes between replicated control and ABA-treated. We randomly selected 12 representative long non-coding transcripts. All LncRNAs were significantly up-regulated under ABA treatment based on qRT-PCR (Figure 5), which showed a high degree of consistency between RNA-Seq and qRT-PCR (Figure 5 and Supplementary Table 9).

## Discussion

In the study, the transcriptome data of strawberry developing fruit with exogenous ABA and its inhibitor NGDA treatment were assessed for LncRNA mining and further conjoint analysis of gene co-expression network and gene ontology with coding RNAs. We discovered 14,552 LncRNAs and 3,595 novel transcripts using the transcriptome dataset, including some previously unidentified functional genes such as FHY3/FAR1. FHY3 is a transcription factor that plays an essential role in the response of plants to light and in photomorphogenesis. *FHY3* gene is derived from a part of the transposon sequence (transposase coding gene) (Tang et al., 2012). Compared with the previous LncRNA identification on diploid strawberry, *F. vesca* and octoploid strawberry (Kang and Liu, 2015; Edger et al., 2019), we first identified hormone-responsive LncRNAs and further inferred their potential biological function by weight-coexpression network construction.

Besides, we further discovered that 8,617 transposon-derived LncRNAs (TE-LncRNAs) are derived from transposable elements (transposon) in the genome. The transposon is a DNA sequence that can move or copy itself to a different position in the genome. It has a vital role in evolution because it can change a cell’s genetic identity and the size the genome (Lisch, 2013). Moreover, in the process of evolution, transposons could vary and transform into sequences with other biological functions, such as miRNA or LncRNA. More than half of the identified LncRNAs derived from TE in this study. The result implied that TE might play an essential role in strawberry genome polyploidization and transmission. Only a few reports about strawberry transposon prediction emerged (Edger et al., 2019). Hence the function of transposon and transposon-derived genes need to be analyzed intensively in future studies.

The present study also investigated how exogenous ABA and its inhibitor influence the LncRNAs expression in the strawberry fruit ripening process. A total of 412 LncRNAs (282 TE-LncRNA) and 382 LncRNAs (231 TE-LncRNA) were identified with significantly differential expression responding to ABA and inhibitor (NGDA), respectively. The co-expression network analysis had 27 modules constructed, all of them containing LncRNAs and coding genes. This result implied that ABA regulates strawberry fruit ripening *via* a complicated mechanism with intensive interaction and cooperation between LncRNAs and coding genes. As reported by Liao et al. (2018) in diploid strawberry (*F. vesca*) the homeostasis is controlled by the expression of *FveCYP707A4a* (cytochrome P450 monooxygenase). Cytochrome P450 monooxygenase is the key enzyme of ABA degradation. There were five homologous of FveCYP707A4a identified: FxaC_7g36210.t1, FxaC_5g00020.t1, FxaC_5g36690.t1, FxaC_8g11790.t1, and FxaC_6g40060.t1. The expression levels of FxaC_7g36210.t1 and FxaC_5g00020.t1 increased 5 days after ABA injection, but FxaC_5g00020.t1 was downregulated at ABA 8-day. FxaC_7g36210.t1 belonged to the module “MEtan,” which correlated with ABA 5-day and had 697 members. There were 52 LncRNAs in “MEtan” module when TE-LncRNA MSTRG.27422.1 played an important node in the network. On the other hand, gene FxaC_9g05310.t1 encoding an isoform of cytochrome P450 monooxygenase, its expression level was significantly induced at ABA 5- and 8-day. This gene belonged to module “MEblack” which had the highest association with ABA 8-day. This module had 1,684 members, among which 126 LncRNA were included. LncRNAs such as FxaC_14g11020, MSTRG.5872.1, and MSTRG.5719.1 were significant nodes in this network. The upregulated expression level of cytochrome P450 monooxygenase coding genes was possibly induced by the ABA injection, which disturbed the ABA homeostasis.

Interestingly, the expression level of ABA1s and NCDEs was elevated after exogenous ABA injection, but not NGDA. ABA1 (zeaxanthin epoxidase) and NCDE (9-cis-epoxycarotenoid dioxygenase) are critical enzymes of the endogenous ABA biosynthesis pathway. This phenomenon implied that the exogenous ABA injection might improve the endogenous ABA biosynthesis, accelerating the ripening process.

The gene ontology analysis performed in the study did not cover all the differentially expressed LncRNAs and coding genes but selected those in the modules with the highest association with the treatments. The result was quite remarkable. First, the exogenous hormone and inhibitor injection induced the gene expression responding to chemicals, which was understandable. Furthermore, all the treatments also induced the response process to abiotic stimulus. It might also indicate the general influence of exogenous chemical injection and collateral damage. ABA is an essential hormone regulating plants tolerant to abiotic and biotic stress (Lee and Luan, 2012; Danquah et al., 2014). It is well known that ABA induces the closure of stoma to reduce evaporation (Hsu et al., 2021; Movahedi et al., 2021). Hence in the GO analysis at ABA treatment, the module contained genes responding to heat and temperature. This result implied those genes involved in stress tolerance might play some roles in the fruit-ripening process as well. Some studies reported that ABA could downregulate photosynthesis (Leng et al., 2021; Park et al., 2021), and in the analysis, the pathways of response to light (stimulus and intensity) were significantly enriched. It is well-known that light could induce the fruit ripening, hence present result showed ABA and its corresponding genes and LncRNAs could be a part of the mechanism. Furthermore, this experimental phenomenon indicated that the exogenous hormone injection had induced and activated the massive number of genes of multiple pathways; those involved in the fruit ripening process and or even more genes activated were relevant to stress-tolerance relevant pathways.

Comparing with exogenous ABA and NDGA treatment could reveal the transcriptome profile variation when the endogenous ABA level decrease in the ripening fruit. NDGA is also an exogenous chemical. It can effectively inhibit 9-cis-Epoxycarotenoid dioxygenase, the key enzyme of ABA biosynthesis (Han et al., 2004). Therefore, compared with CK, the NDGA-treated fruit ought to have a lower ABA level, which would delay its ripening progress. It could explain the GO analysis result of NGDA treatment showing the biochemical pathways of organic acid metabolism (Hwang et al., 2019), phenylpropanoid metabolism (Crizel et al., 2020; Pott et al., 2020), small molecule metabolism, response to disaccharides and lipid metabolism significant enriched. The hypothesis is that NDGA treatment decreases the ABA level in the developing fruit, and affects its nutrient compound biosynthesis and deposition, including organic acid, sugar, lipids (aroma), phenylpropanoid, and other small molecules. On the other hand, the photosynthesis, light-harvesting, response to light density pathways, etc., were also enriched in the gene-network module of NDGA treatment. We deduced that it could be a partial result of the delayed fruit ripening (green fruit) and postponed the fruit-color transmission (Warner et al., 2021).

## Conclusion

In the study, the transcriptomic data of octoploid strawberry developing fruit treated with exogenous ABA and its inhibitor NDGA were used for LncRNA mining and co-expression analysis. The result provides a massive amount of LncRNA, especially transposon-derived LncRNA (TE-LncRNA) in strawberries, and identified those expressed in fruit and the part which was ABA-responsive, potential fruit ripening, and abiotic stress relevant. The LncRNAs and the co-expression modules would serve as promising references and databasess for further studies on fruit ripening in strawberries or other Rosaceae crops, and ABA signal pathways.

## Data Availability Statement

Publicly available datasets were analyzed in this study. RNA-seq data under treatments can be found in the publicly accessible NCBI Sequence Read Archive (SRA) Database under the accession number PRJNA338879.

## Author Contributions

YL and FH designed the study. XC, CW, BH, ZW, and YZ collected plant materials, performed the experiments, and carried out the data analyses. XC, FH, and YL wrote the manuscript. All authors have read and approved the final version of this manuscript.

## Conflict of Interest

The authors declare that the research was conducted in the absence of any commercial or financial relationships that could be construed as a potential conflict of interest.

## Publisher’s Note

All claims expressed in this article are solely those of the authors and do not necessarily represent those of their affiliated organizations, or those of the publisher, the editors and the reviewers. Any product that may be evaluated in this article, or claim that may be made by its manufacturer, is not guaranteed or endorsed by the publisher.

## Footnotes

1
www.rosaceae.org

2
https://github.com/Lyd0527/RNAnorm

## Supplementary Material

Supplementary Table 1 Updated annotation based on RNA-Seq of ABA/NDGA treatments.

Supplementary Table 2 The detail of potential LncRNAs.

Supplementary Table 3 The information of transposon-derived LncRNAs.

Supplementary Table 4 Differentially expressed LncRNAs.

Supplementary Table 5 Modules of co-expression network and their correlation with treatments.

Supplementary Table 6 Genes from modules and their correlation with treatments.

Supplementary Table 7 GO (Gene Ontology) enrichment result.

Supplementary Table 8 Conservation of LncRNA among three related species.

Supplementary Table 9 qRT-PCR primers.
